# Paediatric and perinatal postmortem imaging: mortui vivos docent

**DOI:** 10.1007/s00247-014-3155-1

**Published:** 2014-09-20

**Authors:** Owen J. Arthurs, Rick R. van Rijn

**Affiliations:** 1 Department of Radiology, Great Ormond Street Hospital for Children NHS Foundation Trust, Great Ormond Street, London, UK WC1N 3JH; 2 Institute of Child Health, University College London, London, UK; 3 Department of Radiology, Emma Children’s Hospital - Academic Medical Centre, Amsterdam, The Netherlands; 4 Department of Forensic Medicine, Netherlands Forensic Institute, The Hague, The Netherlands

The classic autopsy, although widely recognised as the gold standard in postmortem diagnosis, has shown a steady worldwide decline in the past decades [1]. A large literature review of adults showed that autopsies could reveal a major error rate from 8.4% to 24.4% and a class I error rate from 4.1% to 6.7% [2]. At the other end of the age spectrum, an Irish study in a neonatal intensive care unit showed that unsuspected or unconfirmed clinical conditions were found in 52% (85/164) of cases, and that in 45 cases information of interest to inheritable conditions was found [3]. While there have been other studies with similar findings, the decline in autopsy rates is difficult to prevent. Parental reluctance to accept an invasive autopsy, rather than clinician reluctance to offer one, is the most likely cause for this, and has been attributed to a variety of causes, including religious beliefs, the fear of unethical practices, the fact that the next of kin believe the deceased should be allowed to rest in peace and an emphasis on individual choices made in our society (where the greater good of gaining knowledge may be perceived to be of lesser importance). Finally, economics plays a certain role in this problem, as the conventional autopsy can be both time-consuming and expensive.

In recent years, there has been growing attention in the postmortem use of radiologic imaging techniques in children, either as an adjunct to or a replacement for the conventional autopsy. There have been many publications, mainly (but not solely) aimed at forensic and adult postmortem radiology. In this field, a close collaboration between radiologists and pathologists, each bringing their own special set of skills and knowledge, is essential to take this work forward [4].

The paediatric radiology community is likely to become more involved in cross-sectional postmortem imaging as it gains momentum and acceptability in guiding pathologists in how best to perform an autopsy, and in some cases where imaging is the only form of autopsy performed due to parental wishes.

We believe now is an appropriate time to focus the paediatric radiology community on the current status of the paediatric and perinatal postmortem examination. We had three aims for this special edition of Pediatric Radiology.

The first aim was to invite international leading authors in the field to give an up-to-date consensus view of different aspects of postmortem imaging, in particular discriminating pathology from normal postmortem findings. This includes normal postmortem radiographical, CT and MRI findings, and a discussion paper regarding when foetal postmortem imaging can be most useful.

The second aim was to evaluate what the current challenges and/or barriers are to postmortem imaging around the world. We invited five contributors from different countries to give short descriptions of their views, which we hope you will find stimulating.

Our third aim was to evaluate how a postmortem imaging service could be established and run within different legal confines, and what the future of postmortem imaging in children is likely to hold.

We sincerely hope that this current range of articles on postmortem imaging will not only increase awareness but will also stimulate interest in a new and exciting field of paediatric imaging.
